# Supplementary material for: HIVprotI: an integrated web based platform for prediction and design of HIV proteins inhibitors
Source: J Cheminform. 2018 Mar 9;10:12. doi: 10.1186/s13321-018-0266-y (PMC5845081; doi:10.1186/s13321-018-0266-y)
Supplement: Supplementary file 2 — Additional file 2. Source code of HIVProtI web server. [file 13321_2018_266_MOESM2_ESM.zip › HIVprotI_Source-code/hivprotI/weka-3-6-6/doc/weka/gui/beans/GraphEvent.html]

GraphEvent


---


|  |  |  |
| --- | --- | --- |
| **Overview** \| **Package** \| **Class** \| **Tree** \| **Deprecated** \| **Index** \| **Help** \| **Weka's home** | |  |
| **PREV CLASS**   **NEXT CLASS** | **FRAMES**    **NO FRAMES**     **All Classes** |  |
| SUMMARY: NESTED \| FIELD \| CONSTR \| METHOD | DETAIL: FIELD \| CONSTR \| METHOD |  |


---


## weka.gui.beans Class GraphEvent

```
java.lang.Object
  java.util.EventObject
      weka.gui.beans.GraphEvent
```

**All Implemented Interfaces:**: java.io.Serializable

---

``` public class GraphEvent extends java.util.EventObject ```

Event for graphs

**Version:**
:   $Revision: 1.5 $

**Author:**
:   Mark Hall

**See Also:**: Serialized Form

---

**Constructor Summary**

`GraphEvent(java.lang.Object source,
java.lang.String graphString,
java.lang.String graphTitle,
int graphType)`
  
          Creates a new `GraphEvent` instance.


| **Method Summary** | |
| --- | --- |
| `java.lang.String` | `getGraphString()`             Return the dot string for the graph |
| `java.lang.String` | `getGraphTitle()`             Return the graph title |
| `int` | `getGraphType()`             Return the graph type |

**Methods inherited from class java.util.EventObject**

`getSource, toString`

**Methods inherited from class java.lang.Object**

`equals, getClass, hashCode, notify, notifyAll, wait, wait, wait`

**Constructor Detail**

### GraphEvent

```
public GraphEvent(java.lang.Object source,
                  java.lang.String graphString,
                  java.lang.String graphTitle,
                  int graphType)
```

:   Creates a new `GraphEvent` instance.

    **Parameters:**: `source` - the source of the event: `graphString` - a string describing the graph in "dot" format: `graphTitle` - the title for the graph: `graphType` - the type for the graph


**Method Detail**

### getGraphString

```
public java.lang.String getGraphString()
```

:   Return the dot string for the graph

    :   **Returns:**: a `String` value

---


### getGraphTitle

```
public java.lang.String getGraphTitle()
```

:   Return the graph title

    :   **Returns:**: a `String` value

---


### getGraphType

```
public int getGraphType()
```

:   Return the graph type

    :   **Returns:**: a `int` value


---


|  |  |  |
| --- | --- | --- |
| **Overview** \| **Package** \| **Class** \| **Tree** \| **Deprecated** \| **Index** \| **Help** \| **Weka's home** | |  |
| **PREV CLASS**   **NEXT CLASS** | **FRAMES**    **NO FRAMES**     **All Classes** |  |
| SUMMARY: NESTED \| FIELD \| CONSTR \| METHOD | DETAIL: FIELD \| CONSTR \| METHOD |  |


---
